# Improved Analytical Model for Thermal Softening in Aluminum Alloys Form Room Temperature to Solidus

**DOI:** 10.3390/ma16237358

**Published:** 2023-11-26

**Authors:** Gaoqiang Chen, Xin Liu, Junnan Qiao, Tianxiang Tang, Hua Zhang, Songling Xing, Gong Zhang, Qingyu Shi

**Affiliations:** 1 Department of Mechanical Engineering, Tsinghua University, Beijing 100084, China; liux21@mails.tsinghua.edu.cn (X.L.); qjnqjn@yeah.net (J.Q.); ttx20@mails.tsinghua.edu.cn (T.T.); xingsongling@126.com (S.X.); zhangg@tsinghua.edu.cn (G.Z.); 2 Key Laboratory for Advanced Materials Processing Technology, Ministry of Education, Beijing 100084, China; 3 School of Mechanical Engineering, Beijing Institute of Petrochemical Technology, Beijing 102617, China; huazhang@bipt.edu.cn; 4 Beijing Infrastructure Investment Co., Ltd., Beijing 100101, China

**Keywords:** constitutive model, flow stress, temperature, aluminum alloys

## Abstract

In advanced solid-state manufacturing processes such as friction stir welding, the metal’s temperature ranges from room temperature to the solidus temperature. The material strength in the temperature range is generally required for investigating the mechanical behaviors. In this communication paper, an analytical model is proposed for describing the thermal softening of aluminum alloys for room temperature to solidus temperature, in which the concept of temperature-dependent transition between two thermal softening regimes is implemented. It is demonstrated that the proposed model compares favorably to the well-known Sellars–Tegart model and Johnson–Cook model. The constants of the proposed model for nine typical engineering commercial aluminum alloys are documented.

## 1. Introduction

Aluminum alloys, as lightweight high-strength materials, are widely applied in manufacturing many advanced load-bearing structures in aerospace vehicles, airplanes, automobiles, etc. In advanced metals manufacturing (AMM) processes such as friction stir welding, the in-process temperatures that the metal experiences range widely from room temperature to solidus temperature [1,2,3,4]. The value of material strength is generally required to study the in-process mechanical behaviors. Taking friction stir welding for example, plastic deformation of materials is driven by tools during the welding processes, which drives the surrounding metal to undergo plastic flow. The dependence of material strength on temperature could affect the expansion of the material flow area around the welding tool due to the viscous nature of the metal and shape the in-process mechanical behaviors such as flow velocity. The constitutive model of material, which describes the thermal softening for room temperature to solidus temperature, plays an important role in numerical simulation. Therefore, one of the major concerns in the computational models for AMM processes is the quantitative accuracy of the thermal softening model that describes the reduction of material strength with temperature rising.

Based on this, researchers have proposed various constitutive models for different materials at elevated temperatures, which can be roughly divided into three categories: phenomenological models [5], physics-based models [6], and artificial neural network models [7]. Phenomenological constitutive models are constitutive models established through empirical and semi-empirical equations based on experimental data and process parameters. Widely used empirical models include the Johnson–Cook model [8] and the Sellars–Tegart model [9]. Although the aforementioned models have been established, there is still a lack of practical and accurate models to describe the softening behavior of metals with temperature. Taking the J–C model as an example, the J–C model is commonly used in finite element simulations because it has an affordable number of parameters and is readily fit. However, the fact that the temperature dependence is defined in a simple power form makes it difficult to describe the realistic temperature softening of materials, especially when the temperature becomes very wide [10,11]. On the one hand, existing models cannot meet the accuracy requirements for simulation in advanced manufacturing processes. For example, in numerical models for friction stir welding [12,13,14], empirical softening has been generally adopted to modify the widely-used Sellars–Tegart model to increase its accuracy in representing the softening phenomenon of metal from room temperature to solidus temperature, in order to mitigate the error in predicting the temperature field and material flow field. On the other hand, artificial neural network models are difficult to integrate with simulations, leading to a lack of practicality [15]. Therefore, in recent decades, some researchers have attempted to modify existing models to improve the predictive accuracy of the model. Shen et al. [9] modified the J–C model by replacing the strain term with the Voce softening model and making adjustments to the thermal softening coefficient. The modified J–C model has a reduced absolute error and provides a more accurate description of the constitutive of 6061 aluminum alloy. Wang et al. [8] took the temperature rise into consideration, which was caused by the conversion of impact compression work into thermal energy and incorporated the temperature rise into the J–C model to correct its temperature term. Compared to the traditional J–C model, the modified model can more accurately predict the mechanical effects of 6063-T5 aluminum alloy under high-temperature dynamic conditions. Colegrave et al. [12] assumed a linear softening model in their numerical simulation model for the friction stir welding process, in which a linear decrease of flow stress to zero as the material temperature increased from 450 °C to the solidus is involved. With this softening model, the simulation model indicated a good agreement between the calculated and experimental values. Chen et al. [13] utilized the Sellars–Tegart model to describe the metal’s constitutive and corrected the softening behavior of metals near the solidus by multiplying a temperature-dependent factor in the model. Only with this softening model, did the calculated temperature distribution during the friction stir welding process match well with experimental data, and a relationship between the total heat generation and the rotational speed of the workpiece was derived. Similarly, Su et al. [14] multiplied a temperature-dependent empirical factor to the Sellars–Tegart model to describe the yield strength. Shi et al. [16] studied the formation of void defects in friction stir welding by multiplying the Sellars–Tegart model with an empirical factor related to the solidus. Geng et al. [17] described the high-temperature softening behavior of metals by multiplying a temperature-dependent power empirical factor on the Jackson–Cook model, which is aimed at studying the influence of rotating tools on the formation and microstructure of aluminum and steel lap joints in friction stir welding. It could be found from the previous research that, although the Sellar–Tegart model can effectively describe the metal’s constitutive in the low and medium temperature ranges and is widely used by most researchers, it shows significant discrepancies in predicting the mechanical properties of metal at high temperatures due to rapid softening caused by changes in the microstructure. The empirical methods used by researchers in dealing with the constitutive during high temperatures are different from each other, and the accuracy of the calculation is achieved by adjusting the empirical factor. It has been recognized that the lack of an analytical model for representing the thermal softening behaviors from room temperature to solidus temperature, has limited the accuracy of the numerical model for the AMM processes. Therefore, a new analytical model for accurately predicting the thermal softening of metals is generally expected.

In this communication, a new analytical model for the thermal softening of aluminum alloys from room temperature to solidus temperature is proposed. The accuracy of the proposed model is discussed based on the handbook data and the comparison with the Sellars–Tegart model and the Johnson–Cook model. Finally, the parameters in the proposed model for typical aluminum alloys are determined.

## 2. Materials and Methods

**Figure 1:**
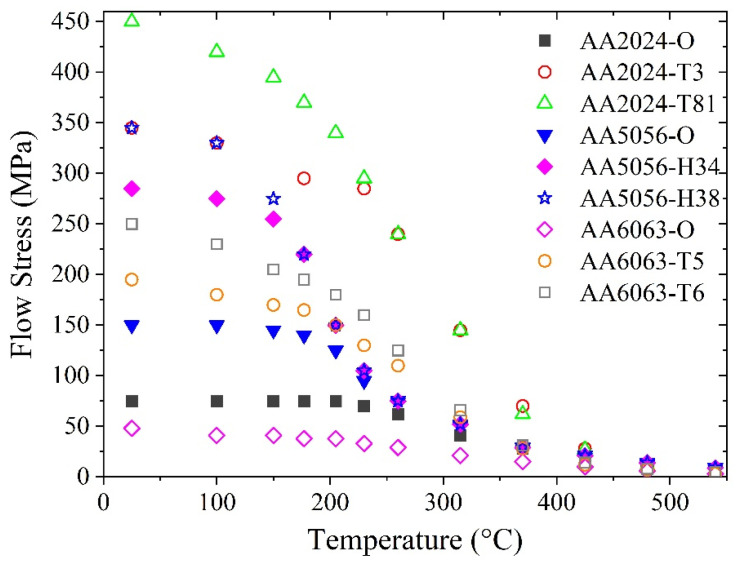
The flow stress of some typical commercial aluminum alloys. The flow stress data is taken as the yield strength in the handbook [18].

Figure 1 shows the flow stress of nine typical commercial aluminum alloys, which is taken from a handbook for aluminum alloys [18]. It could be found from Figure 1 that, the flow stress of the aluminum alloys decreases nonlinearly with rising temperature. It is found that the flow stress temperature curves could be divided into three sections, which are (i) low-temperature section, where the temperature is from room temperature to ~150 °C, (ii) medium-temperature section, where the temperature is from ~150 °C to ~400 °C and (iii) high-temperature section, where the temperature is above ~400 °C. In the low-temperature section, the reduction of flow stress with a rising temperature occurs at a relatively slow rate, because the strengthening components such as dislocations and precipitates, do not significantly change at low temperatures. In the medium-temperature section, the flow stress reduces rapidly due to the precipitates coarsening/dissolution and the reduction in the dislocation density induced by recovery or recrystallization caused by the elevated temperature. In the high-temperature section, the flow stress is approaching zero at a relatively slow rate with temperature rising when the temperature is getting close to the solidus temperature.

## 3. Results

**Figure 2:**
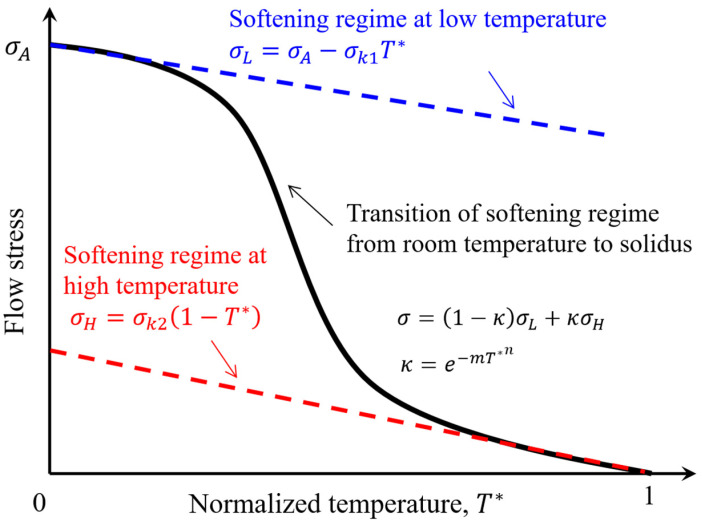
Illustration of the proposed modeling concept for the thermal softening behaviors.

As the flow stress of aluminum alloys decreases nonlinearly with rising temperature, we need to develop an analytical equation that is able to describe the underlying physics. As mentioned above, the decreasing rate of flow stress versus temperature is different in the low-temperature section, medium-temperature section, and high-temperature section. Therefore, we use a linear term to describe the slow decreasing rate in the low-temperature section and high-temperature section, while the rapid decrease in medium medium-temperature section is taken as a nonlinear transition between the low-temperature section and high-temperature section. Figure 2 illustrates the concept in considering the nonlinear thermal softening behaviors in the proposed model. As shown in the figure, we utilize two linear terms to represent the reduction of flow stress versus temperature in the low-temperature section and the high-temperature section. The new thermal softening model is proposed by combining the two linear terms, which is given as,

(1) $$\sigma =\kappa \sigma_{L}+\left(1-\kappa \right)\sigma_{H}$$

where $\sigma_{L}$ denotes the softening regime in the low-temperature section, $\sigma_{H}$ denotes the softening regime in the high-temperature section, and κ is a nonlinear coefficient to combine $\sigma_{L}$ and $\sigma_{H}$. The nonlinear coefficient κ is taken as,

(2) $$\kappa =e^{-mT^{*}{}^{n}}$$

where m and n are coefficients to describe the nonlinear transition between room temperature and high-temperature. $T^{*}$ is the normalized temperature given as,

(3) $$T^{*}=\frac{T-T_{\mathrm{room}}}{T_{\mathrm{solidus}}-T_{\mathrm{room}}}$$

where T is the target temperature, $T_{room}$ and $T_{solidus}$ are the room temperature and solidus temperature, respectively. The value of κ is 1 at room temperature and is getting close to 0 when the temperature approaches solidus. The flow stress in the low-temperature section $\sigma_{L}$, is given as,

(4) $$\sigma_{L}=\sigma_{A}-\sigma_{k1}T^{*}$$

where $\sigma_{A}$ is a constant representing the yield strength at room temperature and $\sigma_{k1}$ is a constant representing the reduction rate for the yield strength. The flow stress in the high-temperature section $\sigma_{H}$, is given as,

(5) $$\sigma_{H}=\sigma_{k2}\left(1-T^{*}\right)$$

where $\sigma_{k2}$ is a constant representing the reduction rate for the flow stress.

Combining Equations (1), (2), (4), and (5), the flow stress in the thermal softening model proposed in this paper is given as,

(6) $$\sigma =\left(\sigma_{A}-\sigma_{k1}T^{*}\right)e^{-mT^{*}{}^{n}}+\sigma_{k2}\left(1-T^{*}\right)\left(1-e^{-mT^{*}{}^{n}}\right)$$

where $T^{*}$ is the normalized temperature by Equation (3), $\sigma_{A}$ is flow stress at room temperature, $\sigma_{k1}$, $\sigma_{k2}$, m and n are constants.

For comparison purposes, we use the model in the literature to discuss the accuracy and the rationality of the proposed model. The two widely applied analytical models of thermal softening of metal are the Sellars–Tegart model and the classic Johnson–Cook model. In the Sellars–Tegart model [19], the flow stress is given as,

(7) $$\sigma =\sigma_{R}\sinh^{-1}\left[\left(\frac{\dot{\varepsilon }}{A}\exp(\frac{Q}{RT}\right){)}^{\frac{1}{n^{\prime }}}\right]$$

where $\dot{\varepsilon }$ is strain rate, Q is a material constant which is deformation activation energy, R is gas constant, T is temperature in Kelvin and $\sigma_{R}$, A, and $n^{\prime }$ are material constants. The strain rate $\dot{\varepsilon }$ is taken as 0.001/s because only the thermal softening is focused. In the Johnson–Cook model [20], the flow stress is given as,

(8) $$\sigma =\sigma_{A}\left(1-T^{*}{}^{r}\right)$$

where $\sigma_{A}$ is flow stress at room temperature, r is the coefficient, and $T^{*}$ is normalized temperature.

The parameters in the proposed model, Sellars–Tegart model, and Johnson–Cook model are determined by minimizing the root mean squared error (RMSE) between the experimental data and the predicted data. The RMSE between the measured and the predicted values of flow stress were calculated by,

(9) $$RMSE=\sqrt{\frac{1}{N}\sum {\left(\sigma_{p,i}-\sigma_{m,i}\right)}^{2}}$$

where N is the number of data points in the dataset, $\sigma_{m,i}$ represents each of the measured flow stress in the dataset and $\sigma_{p,i}$ represents each of the predicted flow stress in the dataset. The determined parameters of the proposed model, the Sellars–Tegart model, and the Johnson–Cook model are given in Table 1 and Table 2.

## 4. Discussion

**Figure 3:**
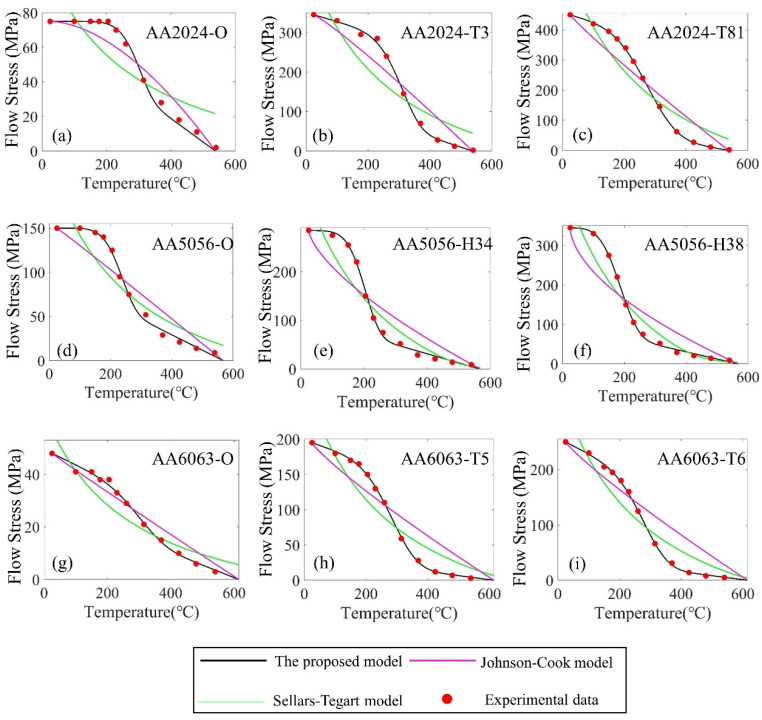
Comparison of the accuracy between the proposed model, Johnson–Cook model, and Sellars–Tegart model fornine types of commercial aluminum alloys, which are (**a**) AA2024-O, (**b**) AA2024-T3, (**c**) AA2024-T81, (**d**) AA5056-O, (**e**) AA5056-H34, (**f**) AA5056-H38, (**g**) AA6063-O, (**h**) AA6063-T5 and (**i**) AA6063-T6.

Figure 3 shows the comparison of predicted flow stress by using the proposed model, the Sellars–Tegart model, and the Johnson–Cook model. Table 3 shows a summary of the average absolute errors of the proposed model, the Johnson–Cook model, and the Sellars–Tegart model. It can be seen from Table 3 that the proposed model has a maximum average error of 11.2%, with most of the errors being below 10%. In contrast, the Sellars–Tegart model and the Johnson–Cook model shows much larger errors, with a maximum average error of up to 292% and a minimum error of 26%. The comparison indicates that the proposed model leads to much less error in representing the flow stress as a function of temperature than the Sellars–Tegart model and the Johnson–Cook model does, and thus the proposed model compares favorably to the Sellars–Tegart model and the Johnson–Cook model in the quantitative accuracy. The proposed model includes a transition between two thermal softening regimes, while the Sellars–Tegart model or the Johnson–Cook model includes only one thermal softening regime. As mentioned above, the thermal softening regime at low temperatures is different from that at high temperatures due to the difference in microstructure. That’s why the accuracy of the proposed model is better than that of the Sellars–Tegart model or the Johnson–Cook model, within the temperature range from room temperature to solidus temperature. In this paper, the effect of temperature on the constitutive is only taken into consideration. In further work, we will improve the mathematical model, and consider the influence of strain rate on the flow stress.

## 5. Conclusions

An accurate model for predicting material strength in the temperature ranging from room temperature to the solidus is generally required for modeling advanced solid-state manufacturing processes such as friction stir welding. In this communication, an improved analytical model for describing the thermal softening of aluminum alloys for room temperature to solidus temperature is proposed. The constants of the proposed model for nine typical engineering commercial aluminum alloys are determined and documented. The results show that the proposed model has a relatively small and acceptable average absolute error (with a maximum error of only 11.2%), which is significantly lower than the error values of the other two models (with a minimum error of 26% and a maximum error of 292%). It is demonstrated that the proposed model compares favorably to the well-known Sellars–Tegart model and the Johnson–Cook model.

## Figures and Tables

**Table 1:** Values of the parameters in the proposed model.

Parameter	*m*	*n*	$\sigma_{k1}$/MPa	$\sigma_{k2}$/MPa	$T_{room}$	$T_{solidus}$	$\sigma_{A}$
AA2024-O	63.4	6.7	0.0	70.0	25	538	75
AA2024-T3	24.0	5.8	119.5	133.4	25	538	345
AA2024-T81	10.1	3.8	167.8	92.6	25	538	450
AA5056-O	121.2	5.2	0.00047	94.6	25	568	150
AA5056-H34	169.1	4.8	9.7	101.5	25	568	285
AA5056-H38	66.7	3.7	18.2	98.7	25	568	345
AA6063-O	20.9	4.5	34.3	28.3	25	615	48
AA6063-T5	25.2	4.4	93.1	33.8	25	615	195
AA6063-T6	30.5	4.6	172.3	42.4	25	615	250

Note 1: The solidus temperature $T_{solidus}$ is taken from the handbook [21].

**Table 2:** Values of the parameters in the Sellars–Tegart model and the Johnson–Cook model.

Model	Sellars–Tegart Model					Johnson–Cook Model	
Parameter	A($\times 10^{11}$)	$\dot{\varepsilon }$	$\sigma_{R}$	*r*	*Q*($\times 10^{5}$)	$n^{\prime }$	$\sigma_{A}$
AA2024-O	1.13	0.001	3.79	4.31	3.64	1.751	75
AA2024-T3	1.13	0.001	9.16	1.57	2.63	1.116	345
AA2024-T81	5.61	0.001	6.81	0.81	2.55	0.921	450
AA5056-O	5.35	0.001	5.94	2.33	2.73	1.024	150
AA5056-H34	5.33	0.001	14.66	2.28	2.16	0.674	285
AA5056-H38	4.23	0.001	24.70	2.87	1.97	0.571	345
AA6063-O	5.33	0.001	5	2.28	2.16	1.433	48
AA6063-T5	1.87	0.001	9.84	2.00	2.59	0.972	195
AA6063-T6	1.90	0.001	6.65	1.36	2.39	0.876	250

**Table 3:** Summary of the average absolute errors of the proposed model, Johnson–Cook model, and Sellars–Tegart model.

Model	The Proposed Model	Sellars–Tegart Model	Johnson–Cook Model
AA2024-O	2.041%	112.21%	130.8%
AA2024-T3	6.035%	292.9%	139.6%
AA2024-T81	1.187%	218.7%	214.9%
AA5056-O	9.953%	42.82%	42.87%
AA5056-H34	11.20%	30.89%	75.61%
AA5056-H38	10.77%	26.98%	82.38%
AA6063-O	4.789%	40.43%	26.02%
AA6063-T5	6.242%	106.1%	143.6%
AA6063-T6	4.416%	81.43%	90.24%

## Data Availability

Data are contained within the article.
